# Affective State Influences Retrieval-Induced Forgetting for Integrated Knowledge

**DOI:** 10.1371/journal.pone.0056617

**Published:** 2013-02-18

**Authors:** Christof Kuhbandner, Reinhard Pekrun

**Affiliations:** Department of Psychology, University of Munich, Munich, Germany; University of California, San Francisco, United States of America

## Abstract

**Background:**

Selectively testing parts of learned materials can impair later memory for nontested materials. Research has shown that such retrieval-induced forgetting occurs for low-integrated materials but may be prevented for high-integrated materials. However, previous research has neglected one factor that is ubiquitous in real-life testing: affective state.

**Methodology/Principal Findings:**

We investigated whether affect influences the resistance of integrated materials to retrieval-induced forgetting by inducing neutral, positive, or negative affect immediately before selectively testing previously learned textbook passages containing interrelated facts and concepts. As negative affect is known to promote a detail-oriented local processing style, we hypothesized that experiencing negative affect during testing may decrease the protective effects of integration and lead to reoccurrence of forgetting. By contrast, as positive affect is known to promote a relation-oriented global processing style, we hypothesized that experiencing positive affect may support effects of integration and prevent forgetting. Our findings are consistent with these predictions. No subsequent forgetting occurred when testing memories for integrated text materials in affectively neutral and positive states, whereas forgetting occurred when testing in negative states. A correlation analysis showed that forgetting decreased with higher positive affect, with participants experiencing high positive affect even showing facilitation instead of forgetting.

**Conclusions/Significance:**

These findings indicate that affect can moderate the memory consequences of test taking and suggest that educators should use testing as a tool to improve memory with care.

## Introduction

In educational settings, it is common practice to selectively test parts of previously learned materials in order to measure what students know. Several recent studies have pointed out that tests can also have important functions beyond assessment because testing has been shown to strongly improve later memory for tested materials (i.e., testing effect; see [1] for a review). Based on such findings, it has been recommended that the number of tests in education should be increased as frequent testing may boost achievement at all levels of education [2].

It is also well known, however, that the improved memory for tested materials can come at the cost of impaired memory for nontested materials, a phenomenon called retrieval-induced forgetting (see [3] for a review). Accordingly, there may be a dark side of testing in educational contexts. Fortunately, although the existence of retrieval-induced forgetting has been demonstrated across a broad range of experimental settings and materials, including real-life issues like the formation of impressions [4] or the acquisition of factual knowledge [5], there seems to exist an important exception which often applies to educationally relevant materials: Retrieval-induced forgetting seems not to occur when knowledge is well integrated (e.g., [6]–[8]).

However, the prevention of retrieval-induced forgetting by integration may depend on an important psychological factor that is ubiquitous in settings of learning and achievement: the affective state during test taking. A large body of research shows that affect can influence a wide range of cognitive processes, indicating that experiencing positive or negative affect is accompanied by qualitatively different styles of processing (see [9], [10], for reviews). Specifically, negative affect has been found to promote a detail-oriented local processing style (i.e., focussing on local details at the expense of global relations between the details), whereas positive affect has been found to promote a relation-oriented global processing style (i.e., focussing on global relations at the expense of local details). For instance, individuals experiencing negative affect focus more on local stimulus features of perceptual experience at the expense of gist [11], are less likely to activate concepts associated with given stimuli [12], and show a reduced activation of stereotypic cognitions associated with social categories [13]. By contrast, individuals experiencing positive affect have been shown to find more easily connections between weakly related words [14], to form broader categories [15], and to reactivate intentionally forgotten memories [16].

Such changes in processing style may also influence the resistance of integrated knowledge to retrieval-induced forgetting. Retrieval-induced forgetting is assumed to result from control processes operating as the result of retrieval competition [17]. During the attempt to retrieve some target memories, other related, but irrelevant, memories are also activated due to spreading activation. To allow the selection of target information, related competitors must be inhibited to reduce interference, leading to later forgetting of the inhibited contents. As interconnections between memories reduce retrieval competition (e.g., [18]), there should be little need to inhibit nontarget information when memory contents are highly integrated. However, one major prerequisite for protection by integration is that existing interconnections are indeed activated during retrieval. Such an activation of interconnections may be influenced by affect. More specifically, by promoting a relation-oriented global processing style, positive affect should enhance the activation of interconnections and support protection by integration. By contrast, due to promoting a detail-oriented local processing style, negative affect should reduce the activation of interconnections, thus reducing their protective effects and leading to retrieval-induced forgetting even for integrated materials.

To test these predictions, the present study investigated whether positive versus negative affect influences the occurrence of retrieval-induced forgetting for integrated materials. Participants studied textbook passages containing interrelated facts and concepts about geographic and biological topics. Afterwards, participants performed a first test in which only a subset of the materials was tested. Immediately before that test, neutral, positive, or negative affect was induced. We examined whether affect influenced later recall of nontested materials. We expected to find no retrieval-induced forgetting when initially testing in neutral states, replicating prior work. By contrast, testing in negative states should cause forgetting. For testing in positive states, we speculated that there may even be facilitation of nontested contents instead of forgetting.

## Materials and Methods

### Participants

Seventy-two undergraduate students (60 females; mean age = 23.5, *SD = *5.4) participated in the experiment. Participants were tested in groups of two to five. This research was approved by the ethic’s committee of the University of Munich (LMU), and all participants provided informed written consent.

### Materials

Two articles were used, one contained information about characteristics of the toucan bird, the other was about the history of Hong Kong. The articles were taken from a study by Chan, McDermott, and Roediger [19] and were translated into German. Each of the two articles was approximately 1,900 words long. For each article, 24 questions were used, composing two sets of 12 questions per article. Each of the answers in one set was related to one of the answers in the other set in terms of conceptual similarity or physical proximity, but none of the information included in one set answered questions in the other set (for more details, see [19]).

To induce affect, a neutral, positive, or negative film clip was presented. The clips were drawn from two sets of standardized film stimuli serving the induction of affect (*neutral*: “Weather Forecast”, *positive*: “Harry and Sally”, *negative*: “Lambs”; see [20], [21], for details). To assess the success of affect induction, we used the affect grid [22] which measures current affect on the dimensions of valence (1 = extremely negative, 9 = extremely positive) and arousal (1 = low arousal, 9 = high arousal).

### Design

We used a 3×3 mixed-factorial design with the within-participants factor retrieval status (tested, nontested, control questions) and the between-participants factor of affect induction (neutral, positive, negative). All participants progressed through four phases. In the study phase, participants studied the two articles. In the subsequent affect-induction phase, the neutral, positive, or negative film clip was presented, depending on condition. Twenty-four participants were randomly assigned to each of the three affect conditions. In the following initial test phase, only one of the two studied articles was tested, using only one of the two question sets belonging to the tested article. Due to this procedure, there were three types of questions, all of which were tested in the final test phase: questions which were initially tested (“tested”), questions which were initially not tested, but belonged to the tested article (“nontested”), and questions belonging to the article that was not tested in the initial test phase, which served as control questions (“control”). The assignment of articles and question sets to experimental conditions was counterbalanced across participants.

### Procedure

In the study phase, participants studied each of the two articles for 15 min under intentional learning instructions. Study order of the initially tested and nontested article was randomized. After a 2-min distractor phase (simple geometrical task), the affect-induction phase followed. Depending on the affect condition, participants viewed one of the three film clips. The clips were presented via a projector on a large screen. Before and after the presentation of the clip, current affective state was measured using the affect grid. Directly after affect induction, participants were given the 12 questions from one of the question sets. The questions were presented one at a time in random order for 15 s via a projector. Participants wrote their answers in a booklet and were told not to guess.

After another 10-min distractor phase (unrelated questionnaire), the final test phase commenced. Participants were given all questions from the four sets of questions (48 questions) and were asked to write their answers on a new page in the booklet. The order of presentation was blocked by question set. The two question sets belonging to the same article were always tested successively, and the testing position of the initially tested article (tested and nontested questions) and nontested article (control questions) was counterbalanced within each affect condition across participants. Within the initially tested article, the nontested questions were always presented first to avoid output interference.

## Results

### Mood Manipulation Check

Before affect induction, participants did not differ with respect to their baseline valence and arousal ratings, *P*s >0.14. After affect induction, both valence and arousal differed reliably between conditions, *F*(2, 69) = 22.34, *P*<0.001, *η_p_* ^2^ = 0.39, and *F*(2, 69) = 14.03, *P*<0.001, *η_p_* ^2^ = 0.29, respectively. Planned comparisons revealed that valence in the neutral condition (*M = *5.4) differed significantly from valence in the positive (*M = *7.3) and negative conditions (*M = *4.5), *t*(46) = −4.45, *P*<0.001, and *t*(46) = 2.02, *P* = 0.049, respectively. Compared with the neutral condition (*M = *4.7), arousal was higher in the positive (*M = *6.3) and negative conditions (*M = *6.7), *t*(46) = −3.81, *P*<0.001, and *t*(46) = −5.07, *P*<0.001, respectively. Arousal did not differ between the positive and negative conditions, *t*(46) = −0.89, *P = *0.376.

### Initial Test Phase

Retrieval success in the initial test phase tended to be higher in the neutral (50.7%) and positive conditions (48.6%), compared to the negative condition (42.4%); the difference, however, failed to reach significance, *F*(2, 69) = 0.97, *P = *0.386.

### Final Test Phase

In the final test phase, when testing memory for the initially tested article, nontested questions were always presented before tested questions. To account for output interference, nontested questions were always compared with the first-tested set of questions related to the control article, and tested questions were always compared with second-tested question set. To determine whether taking an initial test enhanced later memory for tested materials, a 2 (question type: tested, control) × 3 (affect: neutral, positive, negative) analysis of variance was conducted. The analysis revealed a significant main effect of question type, *F*(1, 69) = 4.6, *P = *0.036, *η_p_* ^2^ = 0.06. Participants performed better on the tested questions (49.3%) than on the control questions (43.4%), replicating the testing effect. There was no main effect of affect, *F*(2, 69) = 1.6, *P = *0.201, and no interaction between question type and affect, *F*(2, 69) = 0.05, *P = *0.954, indicating that affect did not influence performance for initially tested materials.

**Figure 1:**
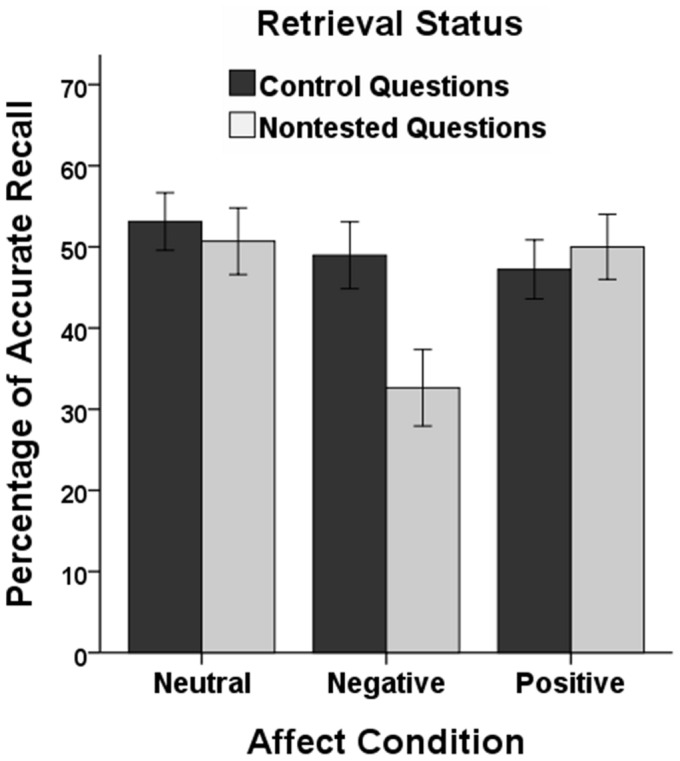
Experimental Results. Percentage of accurate recall in the final test phase for questions on the initially not tested article (control questions) and on nontested contents of the initially tested article (nontested questions) as a function of affective state during initial test-taking (neutral, negative, positive). Error bars represent standard errors of the means.

Figure 1 shows the memory performance for nontested contents of the initially tested article, compared to memory for the control article (for the descriptive statistics for all conditions for both the initial test phase and the final test phase, see Table 1). To examine whether affect influenced later memory of nontested contents, another 2 (question type: nontested, control) × 3 (affect: neutral, positive, negative) analysis of variance was conducted. There was a marginally significant main effect of question type, *F*(1, 69) = 3.26, *P* = 0.075, *η_p_* ^2^ = 0.05, a significant main effect of affect, *F*(2, 69) = 3.33, *P* = 0.042, *η_p_* ^2^ = 0.09, and a significant interaction between both factors, *F*(2, 69) = 3.74, *P* = 0.029, *η_p_* ^2^ = 0.10. Analyzing memory performance separately for each affect condition revealed that in the neutral and positive conditions performance for nontested questions and control questions did not differ, *F*s <0.26, *P*s >0.61. In the negative condition, memory performance was significantly lower for nontested than for control questions, *F*(1, 23) = 11.96, *P = *0.002, *η_p_* ^2^ = 0.34. Whereas memory for control questions did not differ between affect conditions, *F*(2, 69) = 0.65, *P = *0.527, memory for nontested questions was lower in the negative condition compared to the neutral and positive conditions, *F*(1, 46) = 8.39, *P* = 0.006, *η_p_* ^2^ = 0.15, and *F*(1, 46) = 7.88, *P = *0.007, *η_p_* ^2^ = 0.15, respectively.

**Table 1:** Percentage of accurate recall in the initial and final test as a function of question type and affect.

	Initial Test	Final Test					
		Presented First			Presented Second		
Affect	*Tested Questions*	*Control Questions*	*Nontested Questions*	*Amount of Forgetting*	*Control Questions*	*Tested Questions*	*Amount of Enhancement*
*Neutral*	50.7 (4.3)	53.1 (3.5)	50.7 (4.1)	2.4 (4.7)	45.5 (4.2)	51.4 (4.3)	5.9 (5.1)
*Negative*	42.4 (5.2)	49.0 (4.1)	32.6 (4.7)	16.3 (4.7)	37.8 (4.5)	44.8 (4.7)	6.9 (5.2)
*Positive*	48.6 (3.6)	47.2 (3.6)	50.0 (4.0)	−2.8 (5.8)	46.9 (3.2)	51.7 (3.9)	4.9 (3.9)

*Note*. Nontested questions were always presented before tested questions. Amount of forgetting was calculated by subtracting performance on nontested questions from performance on control questions, amount of enhancement by subtracting performance on control questions from performance on tested questions. Standard errors are given in parentheses.

**Figure 2:**
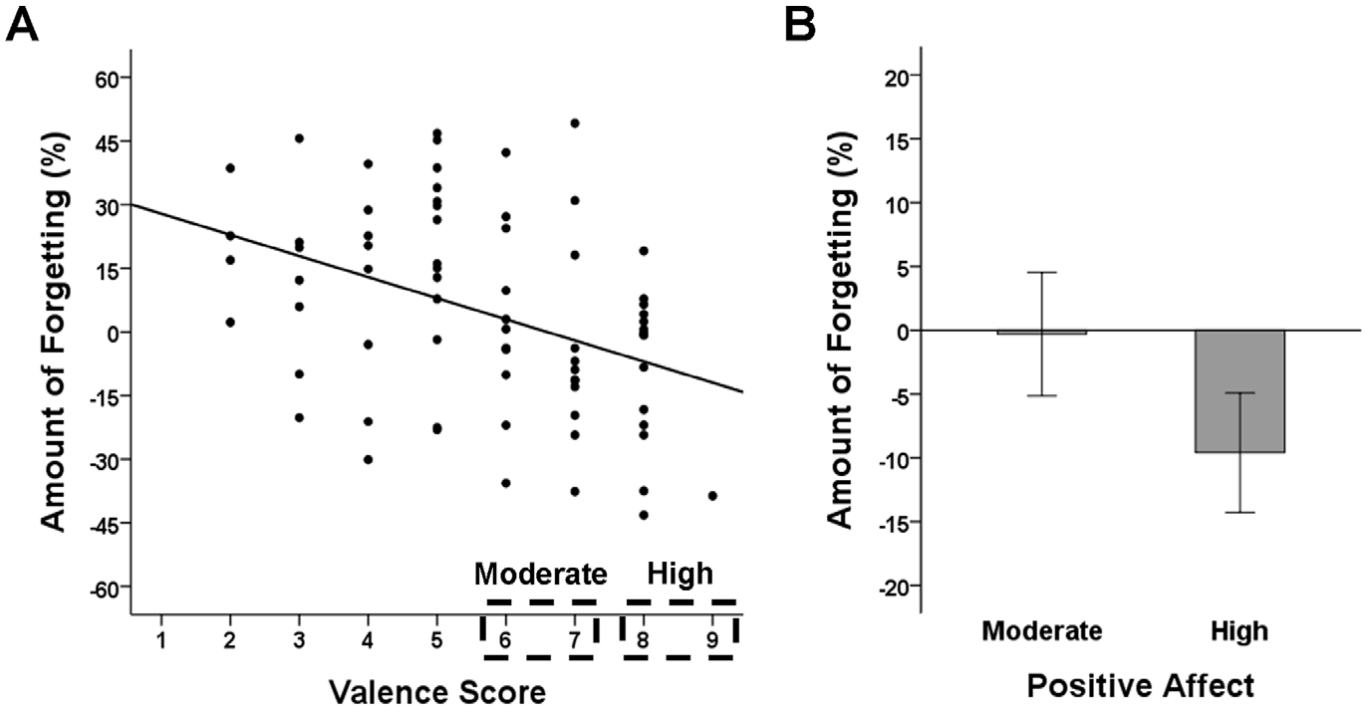
Correlation Analysis. (A) Correlation between amount of forgetting (i.e., decrease in memory performance for nontested questions relative to memory performance for the same questions when used as control questions; larger values indicate a larger amount of forgetting) and post-manipulation valence score. (B) Amount of forgetting as a function of intensity of positive affect (moderate: valence scores 6 or 7; high: valence scores greater than or equal to 8). Error bars represent standard errors of the means.

As indicated by the manipulation check, participants varied in the effects of affect induction. To more thoroughly analyze the influence of current individual affect, we determined the individual amount of forgetting for each participant. To account for the fact that nontested questions and control questions of a participant belonged to different question sets, we determined the individual forgetting rate by subtracting the recall rate for nontested questions from mean recall for the same questions when they were used as control questions for other participants, weighted by a participant’s performance level on his or her own control questions. Correlating the individual forgetting rate with the post-manipulation valence scores revealed a negative relationship, *r* = −0.40, *P*<0.001, indicating that retrieval-induced forgetting increased with higher negative affect and decreased with higher positive affect (see Fig. 2, left panel; correlating post-manipulation valence scores with the uncorrected individual forgetting rate, determined by subtracting recall rates for nontested questions from recall rates for control questions, revealed a similar negative relationship, *r* = −0.34, *P* = 0.003).

Notably, participants experiencing strong positive affect after affect induction (i.e., valence scores greater than or equal to 8; *n* = 16) even showed a reversed memory effect. These participants showed facilitation instead of forgetting (see Fig. 2, right panel), *t*(15) = 2.05, *P* = 0.059. By contrast, post-manipulation arousal scores were uncorrelated with amount of forgetting, *r* = 0.07, *P* = 0.56, indicating that arousal seems not to influence the effects of integration on retrieval-induced forgetting.

## Discussion

The present study demonstrate that affect can influence the resistance of integrated knowledge to retrieval-induced forgetting. When participants were in an affectively neutral state during the initial testing of previously learned integrated textbook passages, no later forgetting of initially not tested contents occurred, replicating previous findings. However, when experiencing negative affect during initial testing, retrieval-induced forgetting occurred. The amount of retrieval-induced forgetting continuously decreased with increased positive affect, with participants reporting high positive affect after induction showing even facilitation instead of forgetting.

The present results are consistent with previous findings indicating that negative affect promotes detail-oriented, local processing whereas positive affect promotes relation-oriented, global processing ([9], [10]). Basically, the resistance of integrated knowledge to retrieval-induced forgetting is explained by the fact that retrieval competition is reduced when memory contents are interconnected, implying that there is little need to inhibit nontarget memories [18]. Concerning the effects of integration for prose materials, a text processing framework has been proposed recently by Chan [8] to explain the occurrence of retrieval-induced forgetting and facilitation for these materials. According to this account, three levels of representation are created when people process prose material (see [23]). The lowest level (surface level) contains the exact wording of the text and is highly transient. The middle level (text-base level) preserves the original meaning in a paraphrased version. At the highest level (mental model level), the pieces of information of the text are stored in a structured way. Pieces of information that are interconnected are incorporated into mental models, which store the newly acquired information as organized multipart memory representations in interaction with preexisting knowledge. When trying to answer specific questions about a text in a memory test, the respective mental model is activated in order to access the target information. Importantly, activated nontarget memories which are part of the same mental model are not treated as competing memories, because they are components of the same multipart memory representation, the parts of which are supposed to be preserved in memory in an integrated way. Accordingly, retrieval competition should be low, and instead of being impaired, nontarget information within the same mental model should benefit from retrieving other contents of the mental model.

According to this framework, a detail-oriented local processing style, as induced by negative affect, and a relation-oriented global processing style, as induced by positive affect, should have opposing effects on the memory consequences of testing. Detail-oriented processing should impair the activation of mental models, because the effectivity of interconnections between memory contents is reduced. This should have two detrimental consequences. First, the protective effect of integration should be reduced because activated nontarget memories are more likely treated as competitors. Second, because accessing target information via mental models is made difficult, the pieces of information of the text are activated in a more unstructured way via the text-base level and the global context of the text, which should generally increase the likelihood of activating nontarget memories. Accordingly, negative affect should not only decrease the beneficial effects of integration, but also increase retrieval competition and thus promote retrieval-induced forgetting. By contrast, a relation-oriented processing style should facilitate the activation of mental models, because such a style facilitates the activation of interconnections between memory contents. This should increase the protective effects of integration, implying that the activation of nontarget items in not counteracted by retrieval inhibition. Accordingly, positive affect should increase the beneficial effects of integration, and when positive affect is high enough, memory for nontarget items may even be facilitated.

Whereas a clear effect of positive affect was evident in the correlation analysis, there was only a small and non-significant trend towards better memory performance for initially not tested contents when comparing memory performance between the neutral and positive conditions. The lack of a similar group-level difference between the experimental neutral and positive conditions may reflect the fact that the establishment of a truly neutral affective state is often difficult because many people experience rather positive affect in neutral contexts [24]. In fact, in the present study, half of the participants in the neutral condition rated their affective state after affect manipulation slightly, or even more than slightly, positive. Accordingly, relation-oriented global processing may also have been a predominant processing mode in the neutral condition, implying that facilitation may have already reached ceiling in the neutral condition (see, e.g., [10], for related arguments). Indeed, weaker effects of positive (compared with negative) affect on the consequences of testing were also observed in a previous study where low-integrated learning materials were used ([25]; see below for details). Thus, negative emotions seem to have more impact on the effects of testing than positive emotions, although this issue warrants further research in which the intensity of the induced emotions is systematically varied. Furthermore, facilitation seems generally hardly to occur in the retrieval-practice paradigm, at least for shorter delays, even with very highly interconnected materials [7]. Interestingly, recent findings suggest that stronger facilitation effects can occur with longer delays between the initial and the final test ([8], [19]). Thus, another interesting avenue of future research would be to assess the influence of affect on long-term effects of testing.

Our findings show that negative affect enhances forgetting for highly integrated prose materials. In contrast, in a previous study by Bäuml and Kuhbandner [25], it was found that negative affect decreases forgetting when learning materials are low-integrated. Thus, depending on the associative structure of learning materials, it seems that negative affect can either enhance or reduce retrieval-induced forgetting. Indeed, such a material-dependent influence of affect is consistent with the framework described above. When the learning material is not presented in a coherent, integrative manner, but instead as separate, randomly-ordered pieces of information (as in the study by Bäuml and Kuhbandner [25] where exemplars of different categories were presented in random order), then it should be difficult to incorporate pieces of information into an integrative mental model. As a consequence, when trying to retrieve a target memory, activated nontarget memories should more likely be treated as distinct and competing memories, and retrieval inhibition and later forgetting should be increased.

Accordingly, a detail-oriented local processing style, as induced by negative affect, should have different consequences for low-integrated than for high-integrated memories. For high-integrated memories, detail-oriented processing should decrease the beneficial effects of integration, because interconnections between memories are less activated. By contrast, for low-integrated memories detail-oriented processing should decrease the detrimental effects of testing, because competing exemplars are less activated. However, although these predictions are well in line with the differential effects of negative affect in the present study and the study by Bäuml and Kuhbandner [25], a more direct comparison of the influence of affect for low-integrated versus high-integrated materials would be needed and would be an important direction for future research.

The present results may also have implications for educational practice. Based on the finding that initial test-taking can enhance later memory, it has been argued that increasing the number of tests in education should be a promising technique to boost academic achievement [2]. When tests are implemented as low-stakes assessments, this may indeed be a useful tool for improving students’ memory (e.g., [26]) because low-stakes tests do not necessarily elicit negative affect. The situation may be different, however, when stakes are high because intense negative emotions are often involved in high-stakes testing [27]. Beyond the well-known fact that experiencing negative affect often causes people to perform below their actual abilities [28], our findings indicate that negative affect can also have detrimental effects on nontested memory contents, even when learning materials are integrated. Accordingly, it may be concluded that tests should be used cautiously as a tool to improve academic learning. However, one also has to bear in mind that utilizing frequent assessments can benefit learning in other ways (beyond the potentially detrimental effects of testing itself), for example, by encouraging students to study more often. In any case, when the question is whether to use tests as a tool to improve academic learning, it may be a good advice to weigh the benefits against possible detrimental effects.
